# *In situ* reduced gold nanoparticles in PDMS contact lenses for color blindness management†

**DOI:** 10.1039/d4ra08879d

**Published:** 2025-04-22

**Authors:** M. Aravind, Haider Butt, Sajan Daniel George

**Affiliations:** a Manipal Institute of Applied Physics, Manipal Academy of Higher Education Manipal 576104 India sajan.george@manipal.edu; b Department of Mechanical & Nuclear Engineering, Khalifa University of Science and Technology P. O. Box 127788 Abu Dhabi United Arab Emirates; c Centre for Applied Nanosciences (CAN), Manipal Academy of Higher Education Manipal 576104 India

## Abstract

Color vision deficiency, or color blindness, is an ocular condition in which individuals have difficulty distinguishing between certain colors. While there is currently no cure for this condition, various wearables can be used to improve the color perception of those affected. The most common wearables used are color-filtering glasses and lenses, which filter out the problematic wavelengths. The most prevalent form of color vision deficiency is red-green color blindness. In this study, gold nanoparticles were *in situ* reduced onto contact lens material, forming plasmonic contact lenses targeted for red-green color blindness management. The absorption of the plasmonic particles, which peaked at around 533 nm, filtered out specific wavelengths to significantly enhance the color perception of both deuteranopia and protanopia. The study also presented an approach of imaging through the plasmonic lenses, followed by color blindness vision simulation to replicate a colorblind individual's vision. When combined with the Ishihara test, this approach proved to effectively improve color perception with the use of plasmonic contact lenses. The study presents a facile method for creating stable, hydrophilic plasmonic contact lenses to manage color blindness. It also offers a unique way to simulate the impact of color filtering on the vision of individuals with color blindness.

## Abstract 2

Plasmonic contact lenses filter problematic wavelengths, enhancing color perception for individuals with deuteranopia and protanopia. Combining imaging and vision simulation, lenses are proved to be an effective solution for improved color vision.

## Graphical abstract

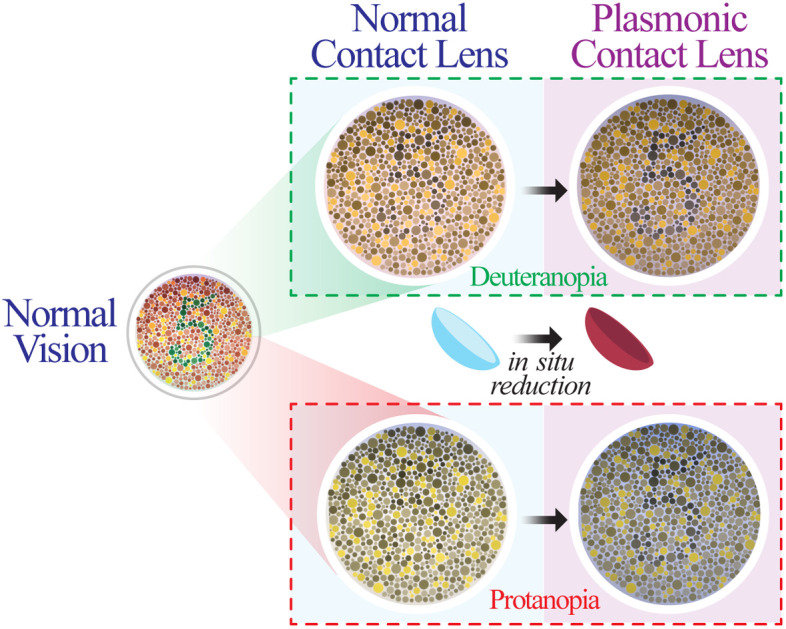

## Introduction

**Fig. 1:**
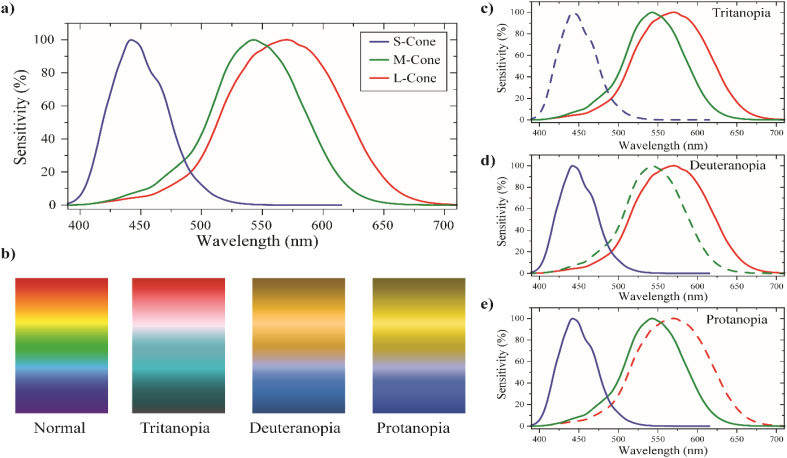
(a) Activation profile of cone cells for normal vision;^23^ (b) representation of how colors are perceived by different types of dichromatism; and (c–e) activation profiles of cone cells for different types of dichromatism.

Color vision deficiency (CVD), or color blindness, is a visual impairment in which individuals have difficulty distinguishing between certain colors. The photoreceptors in the eyes, known as cone cells, are responsible for human color vision. Depending on their responsive wavelength range, cone cells are classified into three types: short, medium, and long wavelength cone cells, also referred to as S-cone, M-cone, and L-cone cells, respectively.^1^ Normal human color vision is trichromatic, wherein all three types of cone cells are present and functioning properly, and the brain receives signals from the corresponding cone cells (Fig. 1a). However, in the case of CVD, the brain receives signals from missing/defective cone cells, resulting in certain colors being indistinguishable. The most common congenital CVDs are inherited on the X chromosome, affecting approximately 8% of males and 0.5% of females. In some populations, the prevalence of CVD in males has reached nearly 15%.^2–4^ The indistinguishability between colors limits the range of activities individuals can perform and often restricts them from working in professions such as the military, aviation, and others.^5,6^

Clinically, CVD patients are classified based on their respective deficiency type. The three different deficiency types of CVD are anomalous trichromacy, dichromacy, and monochromacy. In anomalous trichromacy, one of the photoreceptor cones is defective, classified as tritanomaly (defective S-cone), deuteranomaly (defective M-cone), and protanomaly (defective L-cone). Conditions where one type of cone is missing are classified under dichromacy, with subgroups protanopia (missing L-cone), deuteranopia (missing M-cone), and tritanopia (missing S-cone) (Fig. 1b–e).^7,8^ Monochromacy, the rarest form of CVD, involves the loss of at least two photoreceptor cones. Protans (protanopia and protanomaly) and deutans (deuteranopia and deuteranomaly) are commonly identified as having red-green color blindness, which constitutes 95% of all CVDs, making it the most prevalent form of CVD.^3,9^ The most common technique for diagnosing red-green color blindness is the Ishihara test plates, which is a pseudoachromatic test that utilizes differences in color and contrast to identify whether an individual is color blind or not. Each Ishihara plate consists of a solid circle made up of colored dots that appear in random sizes and colors. Within the pattern, there are dots that form a number or shape visible to those with normal color vision, but invisible or indistinguishable to individuals with red-green color blindness.^10^

Currently, there is no cure available for color blindness, and gene therapy trials are still in development.^11^ As a result, patients often choose methods that can enhance their color perception, such as tinted glasses or lenses.^12–15^ These wearables are customized based on the individual's CVD and are designed to filter out a range of problematic wavelengths, *i.e.*, 520–580 nm for red-green color blindness.^14^ Different methodologies are adopted for the fabrication of color-filtering contact lenses, including infusing nanoparticles or dyes into the hydrogel polymeric network,^7,16,17^ mixing the color-filtering elements into the polymer precursor and mold casting,^14,18^ or 3D printing,^19,20^ creating plasmonic nanostructures.^21,22^ However, the stability of color-filtering elements, such as dyes, within the contact lenses still needs improvement, as they are prone to leakage.^7^ Such leakage of dye often causes undesirable health effects. Moreover, none of the studies so far have presented a quantitative analysis of how the change in the absorbance value in the problematic wavelength range contributes to the improvement in color perception. Such a study is essential, as it provides crucial insights into the minimum amount of color-filtering elements required to improve color contrast, thus avoiding issues with overloading or having an inadequate concentration of nanoparticles or dye in contact lenses.

In this work, gold nanoparticles were reduced *in situ* onto the prepared PDMS contact lenses to filter out the range of optical wavelengths where red-green color blindness patients struggle to distinguish between specific colors. The mold casting, followed by the *in situ* reduction of gold nanoparticles from a gold precursor solution, offers a facile and scalable solution for the preparation of contact lenses for red-green color blindness management. PDMS is a silicon-based material that, in recent years, has been the subject of extensive research in the areas of microfluidics, electronics, and biomedical engineering. Herein, the choice of PDMS for the present study stemmed from the fact that this material is transparent, flexible, biocompatible, and has been used in a wide range of bio-applications. It has high oxygen permeability, making it advantageous for contact lens applications. In characterizing the plasmonic contact lens, its transmission is obtained using a UV/vis spectrophotometer, and the increased reduction time led to increased absorption of the plasmonic particles that peaked at around 533 nm. The plasmonic absorption peak filtered out the problematic wavelength range for red-green colorblind patients. This selective absorption created a darker shade of the affected colors, enhancing the distinction between previously indistinguishable hues, thereby improving overall color perception. As characterized by absorption spectroscopy, the *in situ*-reduced particles were also found to be stable in the contact lens while being stored in a commonly used lens storage solutions for four weeks. Additionally, keeping the plasmonic contact lens in the storage solution was found to retain the inherent hydrophilic nature of the oxygen plasma-exposed plasmonic contact lens for more than three weeks and mitigate the inherent drawback of hydrophobicity in PDMS contact lenses. The plasmonic contact lens's effectiveness in improving color perception was systematically studied against both cases of red-green color blindness *i.e.*, deuteranopia and protanopia. It was found that a plasmonic contact lens significantly enhanced the color perception of individuals with red-green color blindness. This was evident from the improved contrast in the Ishihara test plate, which was typically indistinguishable for those with CVD. The work also presents a unique aspect of how absorbance is related to the color contrast the CVD patient will perceive, by imaging through the plasmonic contact lens, followed by CVD simulation. This study allows us to determine the optimum absorbance required for improving the color perception of CVD patients.

## Experimental

### Materials

Polydimethylsiloxane (Sylgard 184, Dow Corning), gold(iii) chloride trihydrate (Sigma-Aldrich), ethanol (Hayman), contact lens storage solution (Bio true, Bausch and Lomb).

### Preparation of gold-reduced PDMS contact lens

**Fig. 2:**
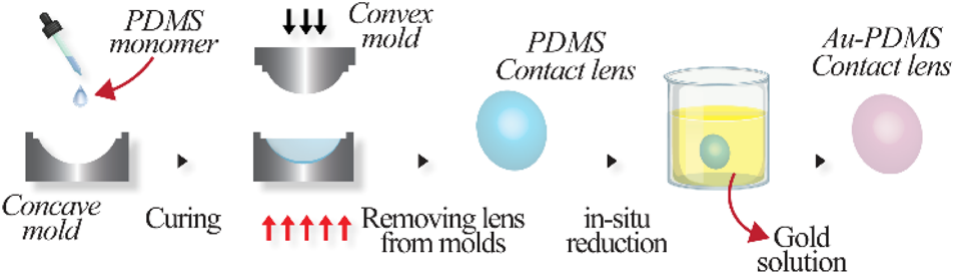
Schematic of the fabrication of gold-reduced PDMS contact lens.

The fabrication of gold nanoparticle-reduced PDMS contact lenses was carried out in a two-step process: the fabrication of PDMS-based contact lenses followed by the *in situ* reduction of gold nanoparticles (Fig. 2). The PDMS contact lenses were fabricated *via* the mold casting method using lathe-made metallic molds. The molds were machined to deliver a contact lens with a cord diameter of 15 mm and a base radius of 8.5 mm. The separation between the convex and concave molds determined the thickness of the contact lens, which, in this case, was ∼80 μm. The PDMS base and crosslinking curing agent were thoroughly mixed in a ratio of 5 : 1 and desiccated to remove air bubbles. Approximately 30 μL of the mixture was added to the concave mold, and the convex mold was pressed against it to form the lens shape and removing the excess. The PDMS was cured at 80 °C for 30 min. After spontaneously cooling to room temperature, the PDMS contact lenses were carefully peeled off from the molds. The PDMS contact lenses were then incubated at room temperature in a 0.01 M ethanolic solution of gold(iii) chloride trihydrate (Sigma-Aldrich) for varying time periods ranging from 6 h to 48 h (denoted as Au-PDMS_6, Au-PDMS_12, Au-PDMS_24, and Au-PDMS_48). The PDMS contact lenses were removed from the gold precursor solution and rinsed with ethanol to remove any excess gold solution. Hydrophilic modification of the contact lenses was performed by exposing them to oxygen plasma at a power of 90 W for 60 s (Femto low-pressure plasma system, Diener Electronic, Germany).

## Results and discussion

**Fig. 3:**
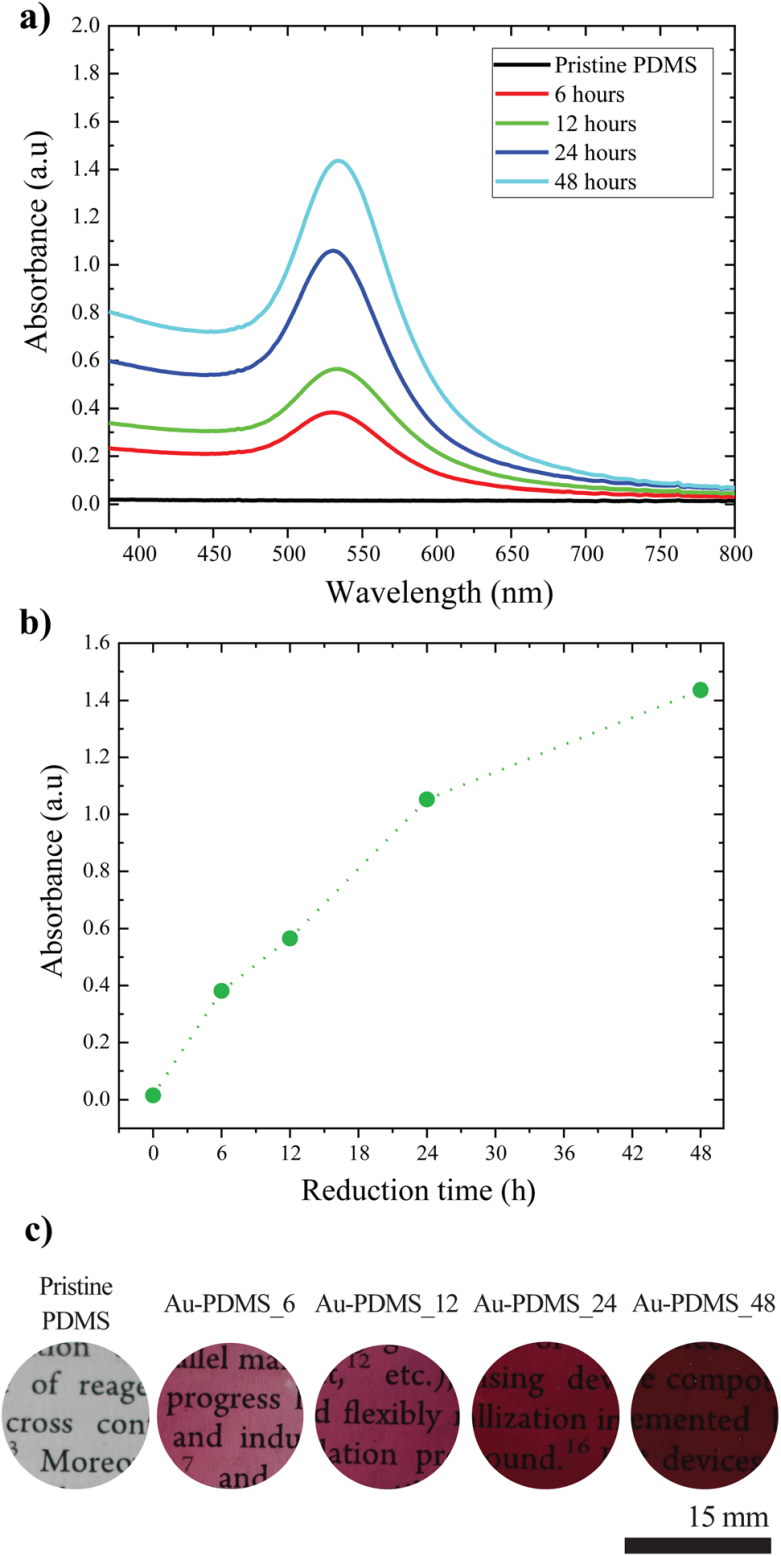
(a) Absorbance spectrum of gold-reduced PDMS with different reduction times; (b) variation in the absorption peak with reduction time; and (c) photographs of PDMS substrates with different reduction times.

The potential of PDMS has already been illustrated in the literature for the fabrication of contact lenses, especially smart contact lenses, with applications in drug delivery and sensing.^24–27^ The crosslinking of PDMS occurs through a hydrosilylation reaction, where silicon hydride (Si–H) groups in the curing agent react with vinyl groups (Si–CH=CH_2_) in the monomer, forming a crosslinked network. The extent of crosslinking and the concentration of unreacted Si–H groups depend on the ratio of the curing agent to the monomer. A higher ratio results in an increased concentration of residual Si–H groups within the cured PDMS. These residual Si–H groups serve as the reducing agents in the *in situ* reduction process.^28,29^ When a PDMS-based contact lens is immersed in a solution of chloroauric acid, the Si–H groups facilitate the *in situ* reduction of gold ions to metallic gold, leading to the formation of gold nanoparticles. This reduction process induces a characteristic red coloration in the PDMS, confirming the successful formation of AuNPs. This phenomenon is consistent with previous studies, which have demonstrated the ability of PDMS to support the *in situ* synthesis of metal nanoparticles *via* its intrinsic reducing properties.^29,30^ The plasmonic absorption of these nanoparticles is monitored using UV-visible absorption spectroscopy. Fig. 3a shows how the absorption spectra of the PDMS film, after being removed from the salt solution, change over different incubation time scales in the HAuCl_4_ solution. The absorption band around 533 nm was due to the well-reported surface plasmon band of gold nanoparticles. As the incubation time increased, the absorbance also increased (Fig. 3b), indicating the formation and increased population of gold nanoparticles. The increase in color of the PDMS substrates corroborated the increase in the population of the gold nanoparticles (Fig. 3c). The consistent absorbance values of the substrate when stored in a commercial contact lens storage solution (Bio true, Bausch and Lomb) for over four weeks indicated the stability of the *in situ* gold-reduced plasmonic contact lenses (Fig. S1†). The *in situ* reduction method offers a simple and scalable fabrication process, unlike the mixing method, which poses issues such as nanoparticle leaching into the ocular environment. The cross-sectional view showed that the gold reduction took place only on the surface and not in the bulk of the lenses, indicating that the process was indeed a surface phenomenon (Fig. S2†).

**Fig. 4:**
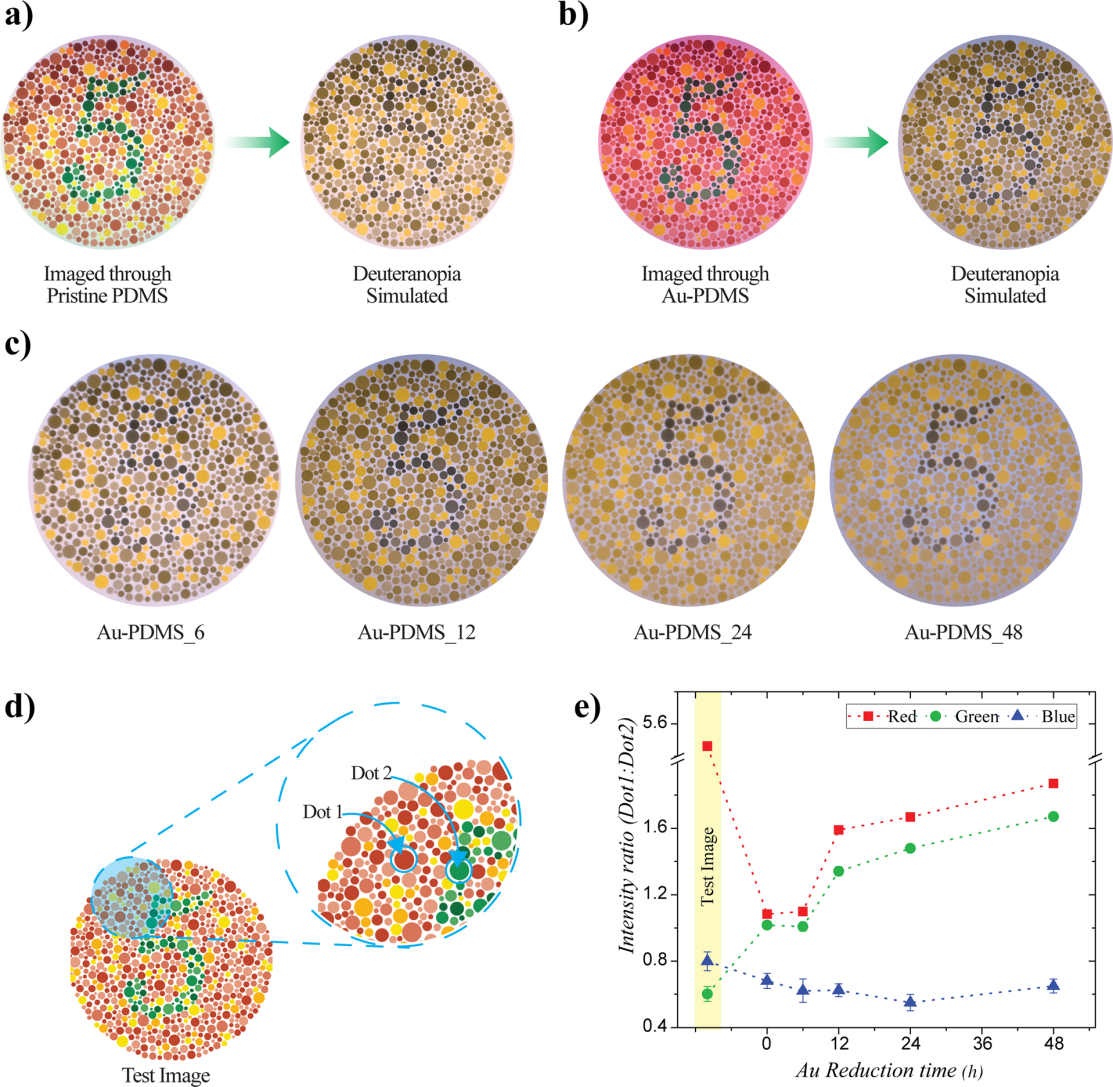
Test image and corresponding deuteranopia-simulated images photographed through (a) pristine PDMS; (b) gold-reduced PDMS (reduction time: 12 h); (c) deuteranopia-simulated images of the test image photographed through gold-reduced PDMS with different reduction times; and (d and e) variation in red, green, and blue channel intensities with different absorbing substrates.

The efficacy of the plasmonic contact lenses was evaluated through imaging and CVD simulation using Ishihara plates. Ishihara is the most commonly used form of pseudoachromatic test by opticians. Ishihara plates consist of dots and random numbers in different colors, which appear indistinguishable or as a single color to individuals with CVD. In this study, we tested the plasmonic contact lenses for two types of red-green color blindness: deuteranopia and protanopia. To mimic CVD vision, we utilized the ‘Daltonize’ plug in the ImageJ software, which simulated different color blindness vision. Color blindness simulation packages, such as Daltonize, simulate different types of CVD primarily through the following process: first, the RGB values of the image pixels are converted into a color space that models human vision. Then, CVD is simulated by reducing colors along a confusion line, making certain hues indistinguishable. Finally, the modified image is converted back to RGB, producing the corresponding CVD simulation. To understand how CVD patients would perceive color through the plasmonic contact lens, a flat piece of plasmonic substrate of the same thickness was attached to a smartphone camera, and photographs were taken in a well-lit room with the distance between the camera and the test image maintained at 0.3 m. These images were then simulated for different types of CVD using ImageJ software for analysis. Trichromatic vision was recorded using a pristine PDMS sample of the same thickness, and the CVD simulation of this image was used as the corresponding CVD reference. Fig. 4a shows the trichromatic and deuteranopia vision, demonstrating how deuteranopia results in an image where distinguishing the pattern from the background is impossible. The Ishihara plate was imaged through the plasmonic substrate, and the corresponding deuteranopia simulation is shown in Fig. 4b. Here, the plate was imaged through a plasmonic substrate with a reduction time of 12 h. Compared to the simulated deuteranopia vision, the image captured through the plasmonic substrate showed improved contrast between the background and the number, resulting in enhanced color perception. The contrast between the two-color regions provided by the plasmonic substrate improved with increasing absorption, which was achieved through longer reduction times, as discussed previously. The test image photographed through plasmonic substrates with reduction times ranging from 6 h to 48 h, followed by deuteranopia simulation, showed the variation in contrast between the two parts of the image (Fig. 4c). To quantify the effect of absorption on changes in color perception, different color components were analyzed. The analysis was performed by choosing two dots in the Ishihara test image with similar brightness—indistinguishable to red-green CVD patients—predominantly red and green in color, as shown in Fig. 4d. The red, green, and blue channel intensities of the two dots were measured using ImageJ software, and the corresponding color intensity ratios were calculated and plotted against the gold reduction time (Fig. 4e). The test image showed a high red and low green intensity ratio, as dot 1 and dot 2 were primarily red and green, respectively. The blue channel intensity ratio remained consistent across all images, as the test image was predominantly composed of red and green colors. Additionally, the blue channel values remained close to those of the original test image, indicating that the plasmonic substrates did not significantly affect the short-wavelength region. This observation was further supported by the absorption spectra, which showed no significant absorption in the blue region. The red and green channel intensities varied with different substrates, and the substrate with zero reduction time represented the deuteranopia vision. As expected, the red and green color intensity ratio was close to unity for the deuteranopia simulation, making it difficult to distinguish between the two colors. Similarly, the color intensity ratio remained nearly unchanged for the plasmonic substrate with a reduction time of 6 h, indicating no significant improvement compared to the deuteranopia vision. However, for plasmonic substrates with a reduction time greater than 6 h, color perception improved noticeably, which was also evident from the photographs. A significant improvement was observed for plasmonic substrates reduced for more than 6 h, and the changes in the red and green channel intensity values further corroborated this enhancement.

**Fig. 5:**
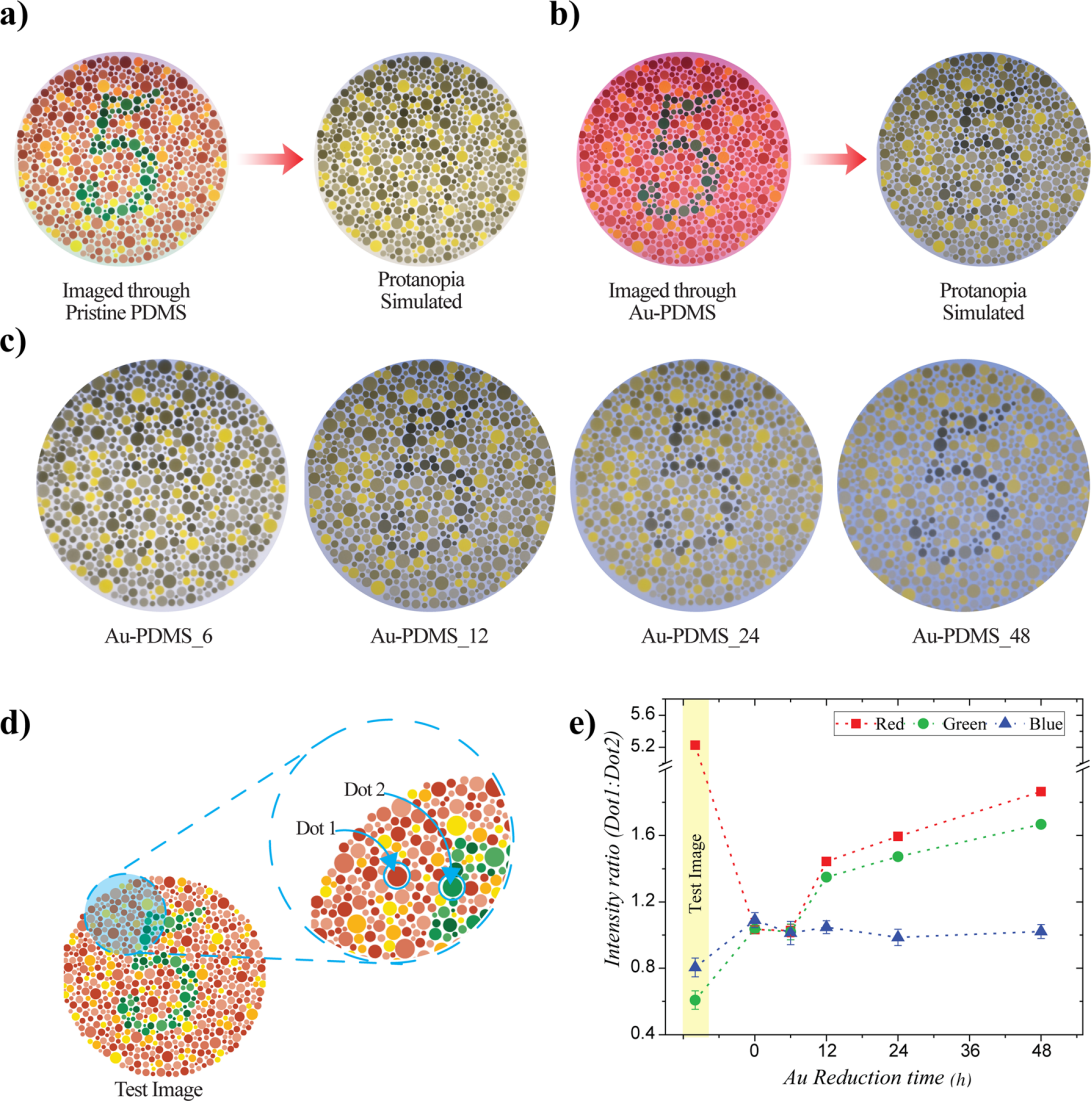
Test image and corresponding protanopia-simulated images photographed through (a) pristine PDMS, (b) gold-reduced PDMS (reduction time: 12 h), (c) protanopia-simulated images of the test image photographed through gold-reduced PDMS with different reduction times; and (d and e) variation in red, green, and blue channel intensities with different absorbing substrates.

Similar to the evaluation of the plasmonic contact lens's efficacy for deuteranopia, we tested its performance against protanopia-type CVD. Following the same methodology, we simulated and compared the test image photograph with images captured through the plasmonic substrates. As demonstrated, protanopia vision made it difficult to identify the number in the Ishihara plate (Fig. 5a), whereas imaging through the plasmonic substrates significantly improved color perception (Fig. 5b and c). The variation in different colors while imaging through the different plasmonic substrates was evaluated in terms of changes in the intensity of the primary colors. As in the previous case, no significant difference was observed in the blue channel intensity, as the image was primarily composed of red and green colors, and the substrate remained transparent to shorter wavelength regions. The intensity ratio was close to unity for the protanopia vision, as expected, and no improvement was observed with plasmonic substrates having a reduction time of 6 h. Increased absorption, achieved through longer reduction times, resulted in better contrast between the two regions (Fig. 5e), making it easier for a protanopia patient to clearly identify the numbers.

To confirm that the changes in color intensity apply throughout the entire image, we conducted the same study using a different set of dots that were indistinguishable to individuals with deuteranopia and protanopia. The study yielded similar results to those discussed earlier, affirming the effectiveness of the method (Fig. S3†). The method was further validated by using different Ishihara plates, and a significant improvement in color perception and the ability to distinguish numbers was observed (Fig. S4†). The simulations were generated using the ImageJ software plugin to corroborate the validity of the approach. Additionally, deuteranopia and protanopia vision were simulated using Coblis (color blindness simulator),^31^ which yielded results similar to those obtained from the ImageJ plugin (Fig. S5†). Furthermore, the imaging and simulation study was conducted by varying the distance between the test image and the camera. The study confirmed that plasmonic contact lenses could provide better color perception for nearby objects. It is important to note that the maximum distance is limited only by the camera's imaging quality and is not a limiting factor of the plasmonic contact lenses themselves (Fig. S6†). The testing method, which combined imaging with CVD vision simulations, provided critical insights into the optimal nanoparticle concentration needed for enhanced color perception, offering an advantage over other published studies (Table S1†).

**Fig. 6:**
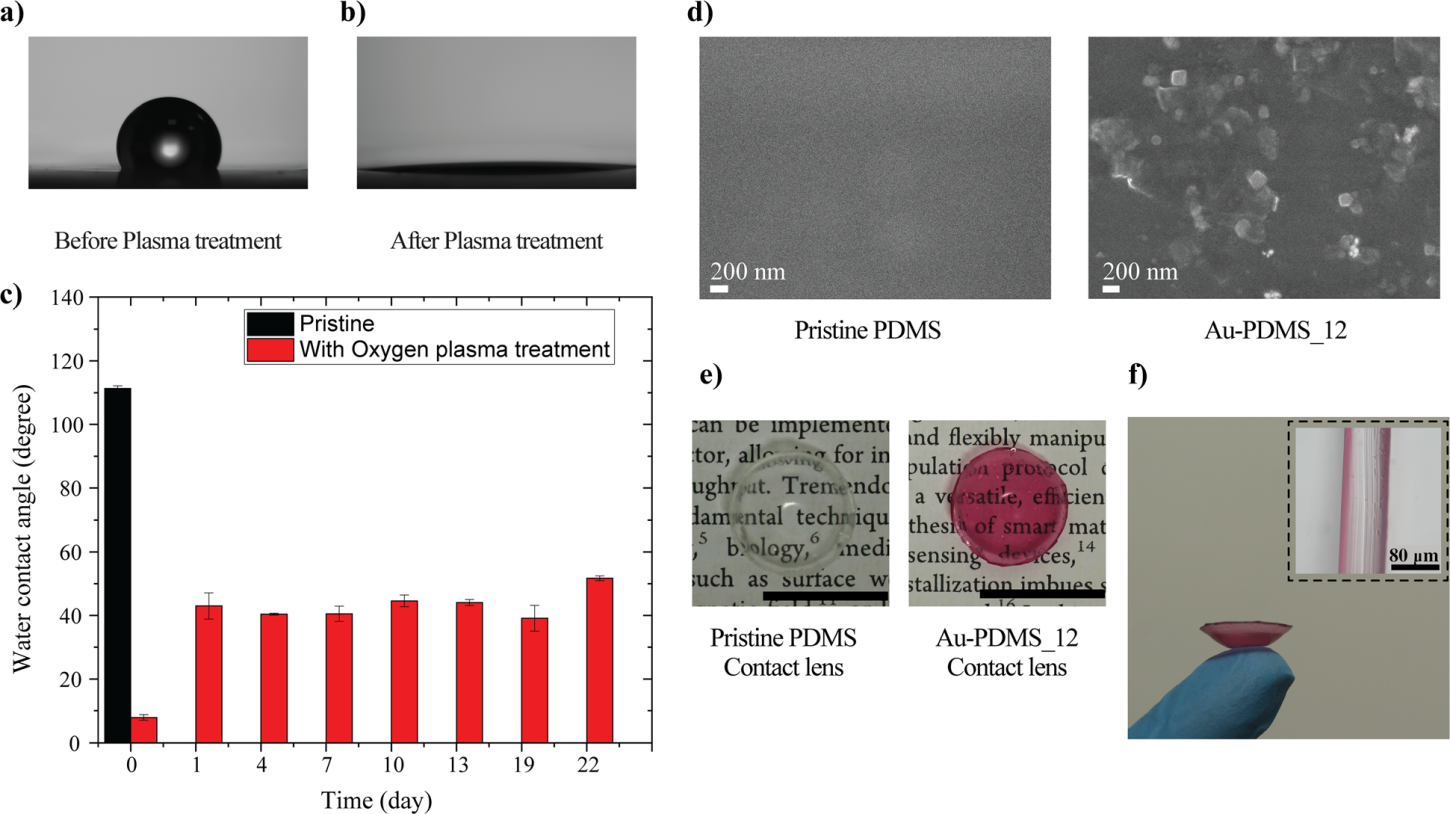
Water contact angle of PDMS substrates (a) before and (b) after oxygen plasma treatment; (c) retention of hydrophilicity in the plasmonic PDMS substrate after oxygen plasma treatment and storage in contact lens storage solution; (d) FESEM images of pristine and nanoparticle-reduced surfaces; (e) photograph of PDMS contact lens before and after gold reduction (scale bar = 15 mm); and (f) cross-sectional image of the plasmonic contact lens.

Wettability is an important factor when considering the properties of contact lenses. Wettability is closely linked to biocompatibility and comfort, as the wettability of the lens determines how stably the tear film spreads on the contact lens surface.^32^ The wettability of the surface was investigated using a commercial contact angle instrument (Holmarc, India) by measuring the static contact angles with a 3 μL drop volume. Data were collected from at least three different positions on each sample. Pristine PDMS was hydrophobic, with a water contact angle of 110 ± 2°, and the *in situ* reduction of gold did not significantly alter the wettability of the surface, which remained hydrophobic (Fig. S7†). To maintain a stable tear film over the contact lens, the surface must be hydrophilic. Subjecting PDMS to exposure from an oxygen plasma system could convert it into a hydrophilic surface, as shown in Fig. 6a and b. This transformation could be leveraged to create a hydrophilic plasmonic contact lens. Although the plasma-treated PDMS surface tended to recover its hydrophobic state due to the migration of low-molar-mass PDMS chains, the hydrophilic properties of the plasma-treated PDMS surfaces could be retained for extended periods when stored in an aqueous solution.^33,34^ The plasma-treated contact lenses were stored in a commercially available contact lens storage solution, and their wettability was monitored. The study indicated that the plasmonic contact lenses retained their hydrophilicity for more than three weeks (Fig. 6c). The FESEM images of pristine and nanoparticle-reduced PDMS substrates with an incubation time of 12 h are presented in Fig. 6d, while the photograph of the fabricated plasmonic contact lenses is depicted in Fig. 6e. Similar to wettability, contact lens thickness is also an important factor, as it affects handling, wearing comfort, oxygen transmissibility, and other factors.^35,36^ Here, the spacing between the convex and concave molds used determined the thickness of the contact lens. The cross-sectional image showed that the fabricated contact lenses had a central thickness of nearly 80 μm, which was comparable to commercially available contact lenses (Fig. 6f).

## Conclusions

Color-filtering contact lenses were successfully created by *in situ* reducing gold nanoparticles onto PDMS contact lenses. The study analyzed the effect of varying the reduction time (6, 12, 24, and 48 h), and it was found that increased reduction time led to greater absorption. The plasmonic absorption band, centered at 533 nm, filtered out the wavelengths problematic for red-green dichromatism in CVD patients. The effectiveness of the plasmonic contact lens in improving color perception was investigated against both types of red-green color blindness, namely, deuteranopia and protanopia. It was found that the plasmonic contact lens significantly enhanced the color perception of individuals with red-green dichromatism. The improvement in color perception was evident from the enhanced contrast in the Ishihara test plate, which was typically indistinguishable for those with CVD. The work also demonstrated a unique aspect of how absorbance was related to the color contrast perceived by CVD patients. This was achieved by imaging through the plasmonic contact lens followed by CVD simulation, which allowed for the determination of the optimal absorbance needed to improve the color perception of CVD patients. The results showed that plasmonic contact lenses with a reduction time of 12 h or more were highly effective in improving the color perception of both deuteranopia and protanopia CVD patients. The PDMS contact lenses, typically hydrophobic, were modified to become hydrophilic through oxygen plasma exposure. The hydrophilicity of the plasmonic PDMS contact lens was found to be maintained for over three weeks when stored in a contact lens storage solution. Additionally, the plasmonic contact lens exhibited stable performance, with consistent absorption spectra, even when stored in the contact lens solution for weeks.

## Data availability

The data are available upon request from the authors.

## Author contributions

Aravind M.: methodology, investigation, data curation, and writing – original draft. Haider Butt: supervision and writing – review and editing. Sajan D. George: conceptualization, supervision, project administration, funding acquisition, and writing – review and editing.

## Conflicts of interest

There are no conflicts to declare.

## Supplementary Material

RA-015-D4RA08879D-s001
