# Supplementary material for: In situ reduced gold nanoparticles in PDMS contact lenses for color blindness management
Source: RSC Adv. 2025 Apr 22;15(16):12765–72. doi: 10.1039/d4ra08879d (PMC12012671; doi:10.1039/d4ra08879d)
Supplement: RA-015-D4RA08879D-s001 [file RA-015-D4RA08879D-s001.pdf]

Supplementary Information (SI) for RSC Advances.
This journal is © The Royal Society of Chemistry 2025

Electronic Supplementary Information

In situ reduced gold nanoparticles in PDMS contact lenses for color blindness management †

*Aravind M.[1], Haider Butt[2], Sajan Daniel George [1,3] **

[1]Manipal Institute of Applied Physics, Manipal Academy of Higher Education, Manipal, India – 576104.
[2]Department of Mechanical & Nuclear Engineering, Khalifa University of Science and Technology, P O Box 127788, Abu Dhabi, United Arab Emirates.
[3]Centre for Applied Nanosciences (CAN), Manipal Academy of Higher Education, Manipal, India – 576104.
Corresponding Author Email: sajan.george@manipal.edu

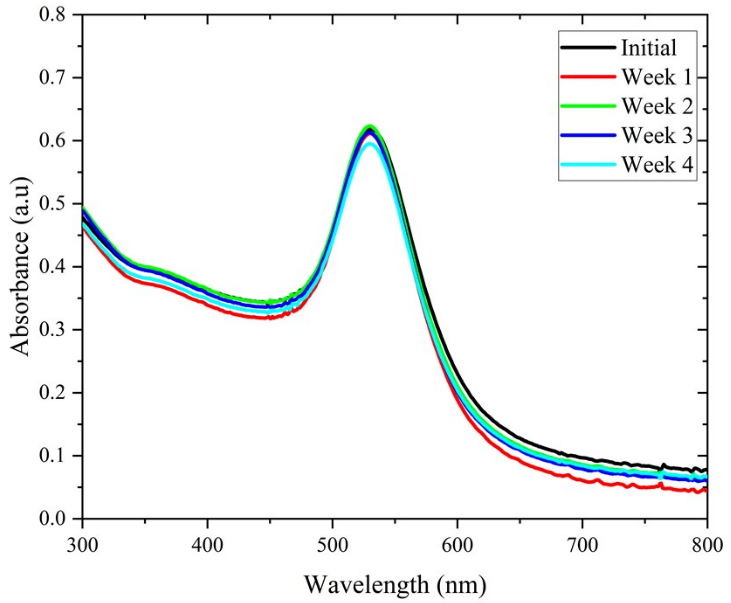

Fig. S1. The stability of the absorbance of plasmonic substrate stored in contact lens storage solution.

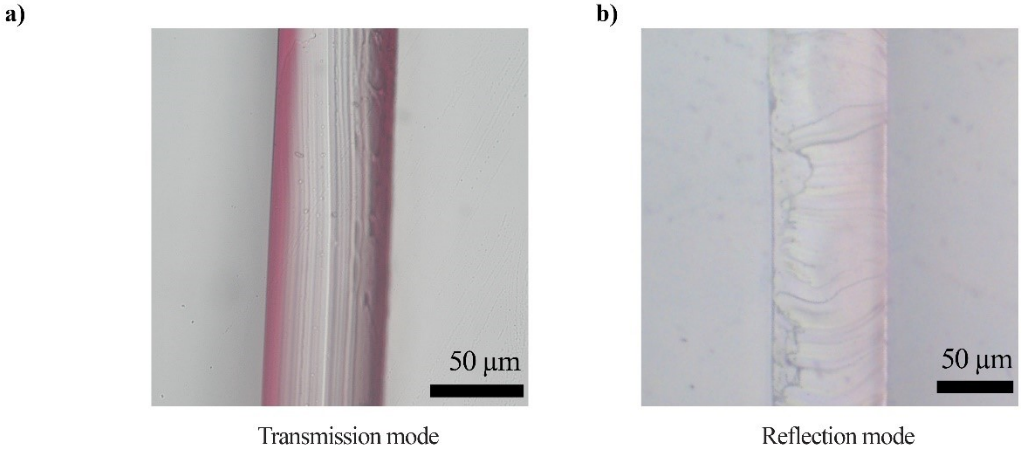

Fig. S2. The cross-sectional image of the plasmonic contact lens captured in both transmission and reflection modes (Reduction time:12 hours).

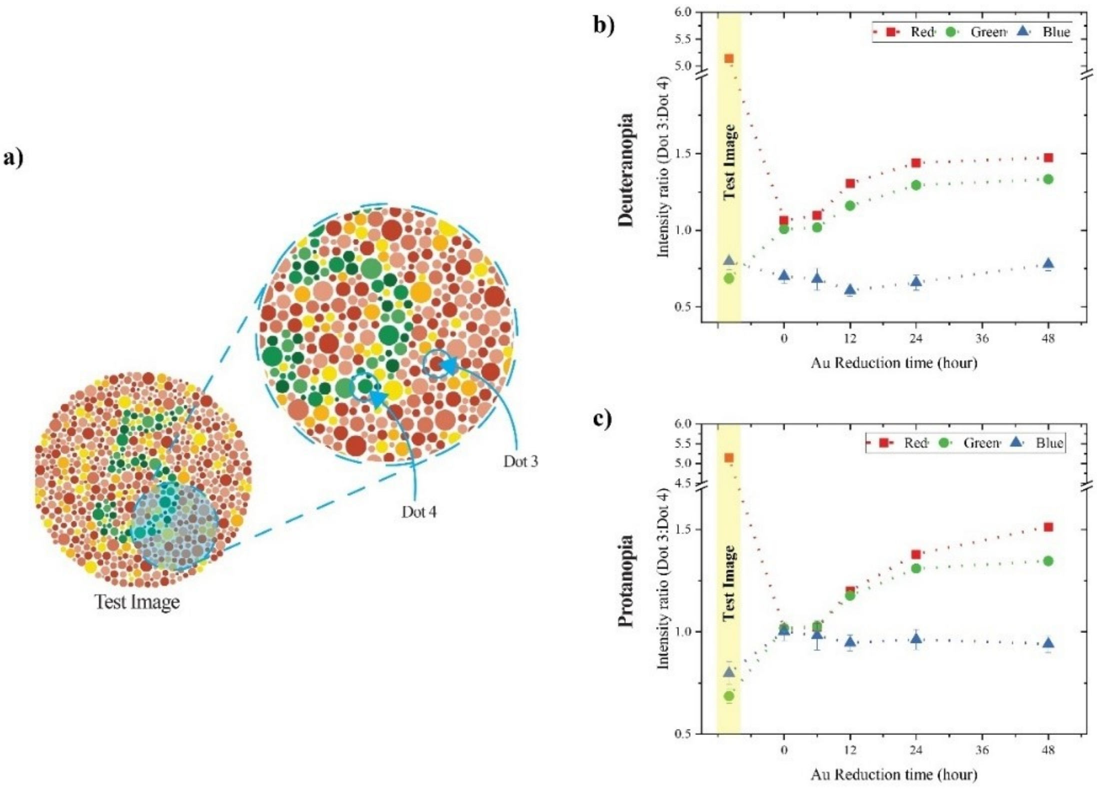

Fig. S3. a) The two points chosen for the study in the test image, b) and c) the variation in red, green, and blue channel intensity with different absorbing substrates for deuteranopia and protanopia respectively.

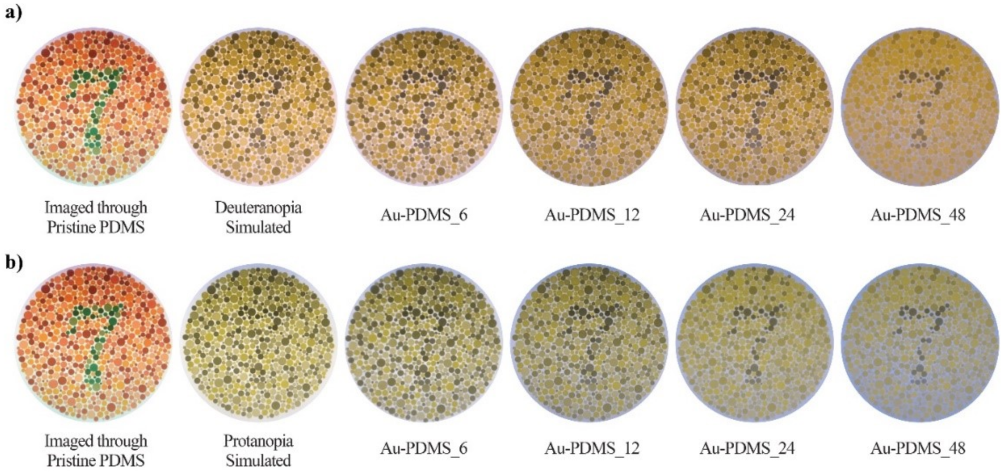

Fig. S4. The CVD simulation generated for images captured through a gold reduced PDMS of different reduction time for a) deuteranopia and b) protanopia.

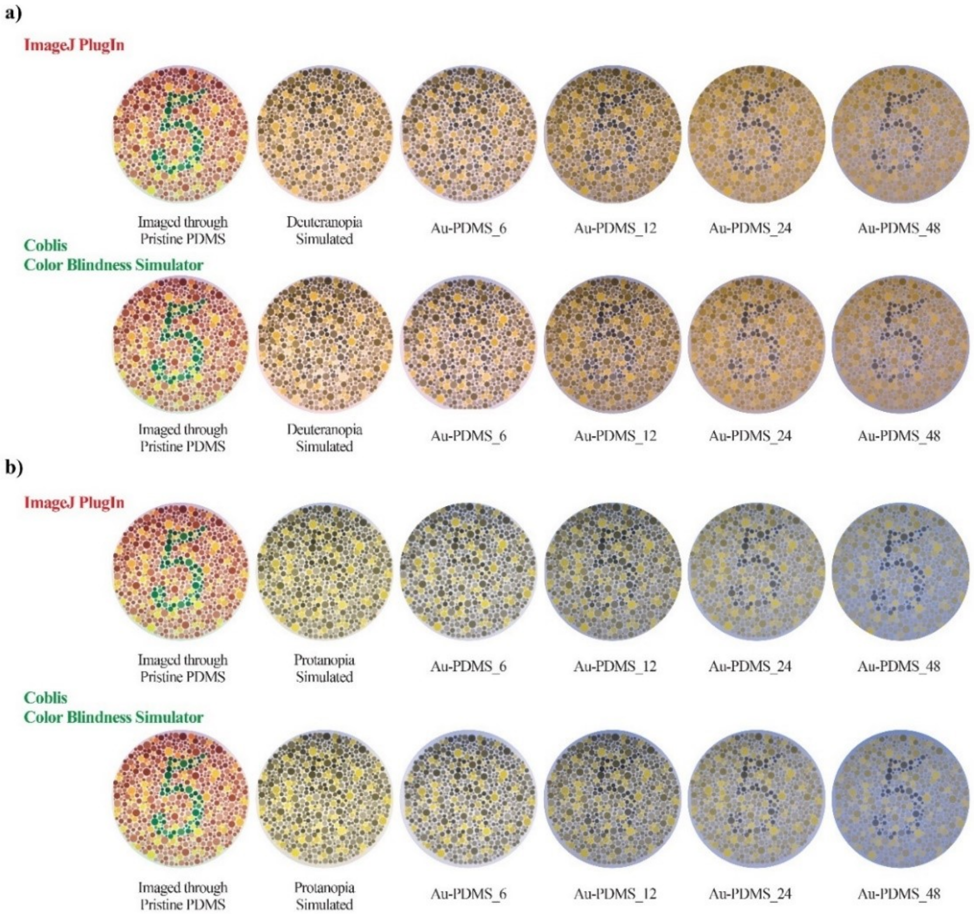

Fig. S5. The CVD simulation generated by using ImageJ plugin and Coblis (Color Blindness Simulator) for a) deuteranopia and b) protanopia.

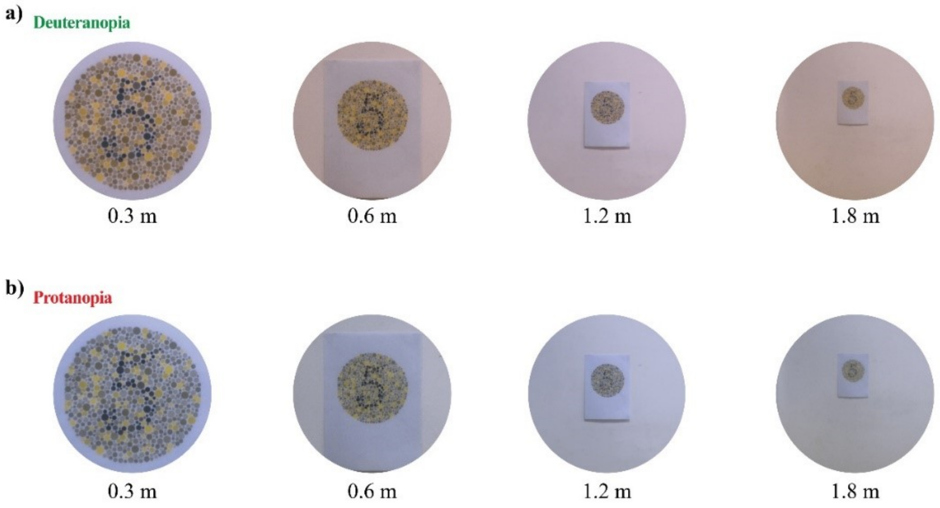

Fig. S6. The CVD simulation generated for different distances between the test image and the camera for a) deuteranopia and b) protanopia imaged through a gold reduced PDMS (Reduction time:12 hours).

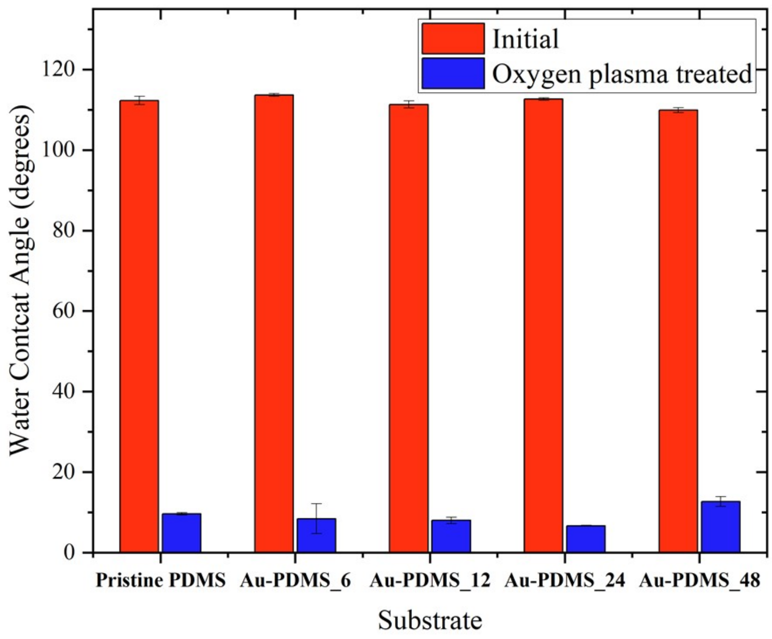

Fig. S7. The water contact angle of plasmonic substrates with different reduction times before and after oxygen plasma treatment.

Table S1. The comparison of literature of using gold nanoparticles for CVD management.

**Paper (Year)**	**Material**	**Fabrication Method**	**Testing Method**
Ahmed E. Salih *et.al. (2021)* [1]	2-hydroxyethyl methacrylate (**HEMA**)	Gold nanoparticles mixed with hydrogel monomer solution.	-
Ahmed E. Salih *et.al. (2022)* [2]	Commercial contact lens **Acuvue**	Breath-in/ breath-out method for infusing gold and silver nanoparticle in commercial contact lens.	-
N. Roostaei *et.al. (2022)* [3]	Polydimethylsiloxane (**PDMS**)	In-situ reduction of gold nanoparticles and soft nano-lithography.	-
N. Roostaei *et.al. (2022)* [4]	Polydimethylsiloxane (PDMS) coating on **Glass**	In-situ reduction of gold nanoparticles.	-
Liya Jacob *et.al. (2024)* [5]	Commercial contact lens **Acuvue**	Breath-in/ breath-out method for infusing gold nanoparticle in commercial contact lens.	-
This study	Polydimethylsiloxane (**PDMS**)	In-situ reduction of gold nanoparticles.	Imaging Ishihara test plates and CVD vision simulations

References

(1) Salih, A. E.; Elsherif, M.; Alam, F.; Yetisen, A. K.; Butt, H. Gold Nanocomposite Contact Lenses for Color Blindness Management. *ACS Nano* **2021**, *15* (3), 4870-4880. DOI: 10.1021/acsnano.0c09657

(2) Salih, A. E.; Elsherif, M.; Alam, F.; Alqattan, B.; Yetisen, A. K.; Butt, H. Syntheses of Gold and Silver Nanocomposite Contact Lenses via Chemical Volumetric Modulation of Hydrogels. *ACS Biomaterials Science & Engineering* **2022**, *8* (5), 2111-2120. DOI: 10.1021/acsbiomaterials.2c00174

(3) Roostaei, N.; Hamidi, S. M. Two-dimensional biocompatible plasmonic contact lenses for color blindness correction. *Scientific Reports* **2022**, *12* (1), 2037. DOI: 10.1038/s41598-022-06089-8

(4) Roostaei, N.; Hamidi, S. M. Plasmonic Eyeglasses Based on Gold Nanoparticles for Color Vision Deficiency Management. *ACS Applied Nano Materials* **2022**, *5* (12), 18788-18798. DOI: 10.1021/acsanm.2c04553

(5) Jacob, L.; Syed, S. S.; Cherumkuzhi, M. S.; Salih, A. E.; Butt, H. Biogenic Gold Nanoparticle-Based Antibacterial Contact Lenses for Color Blindness Management. *Advanced Engineering Materials* **2024**, *26* (5), 2301242. DOI: https://doi.org/10.1002/adem.202301242
